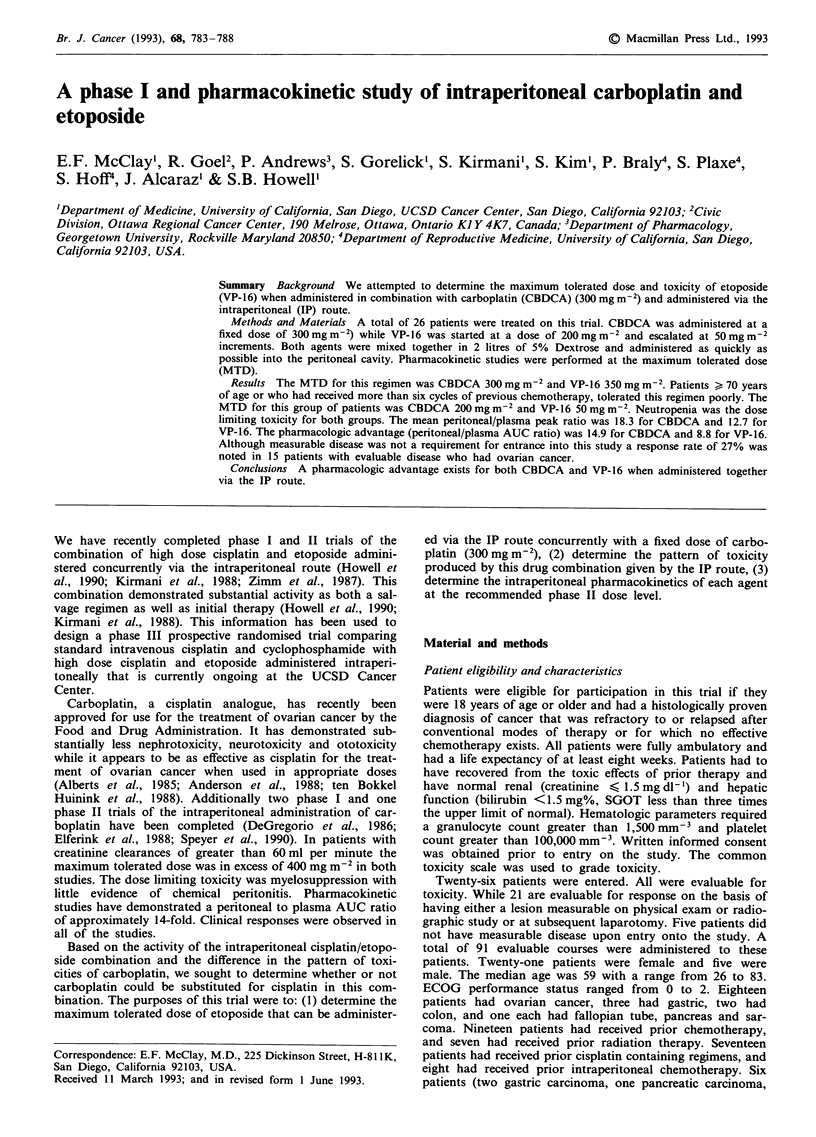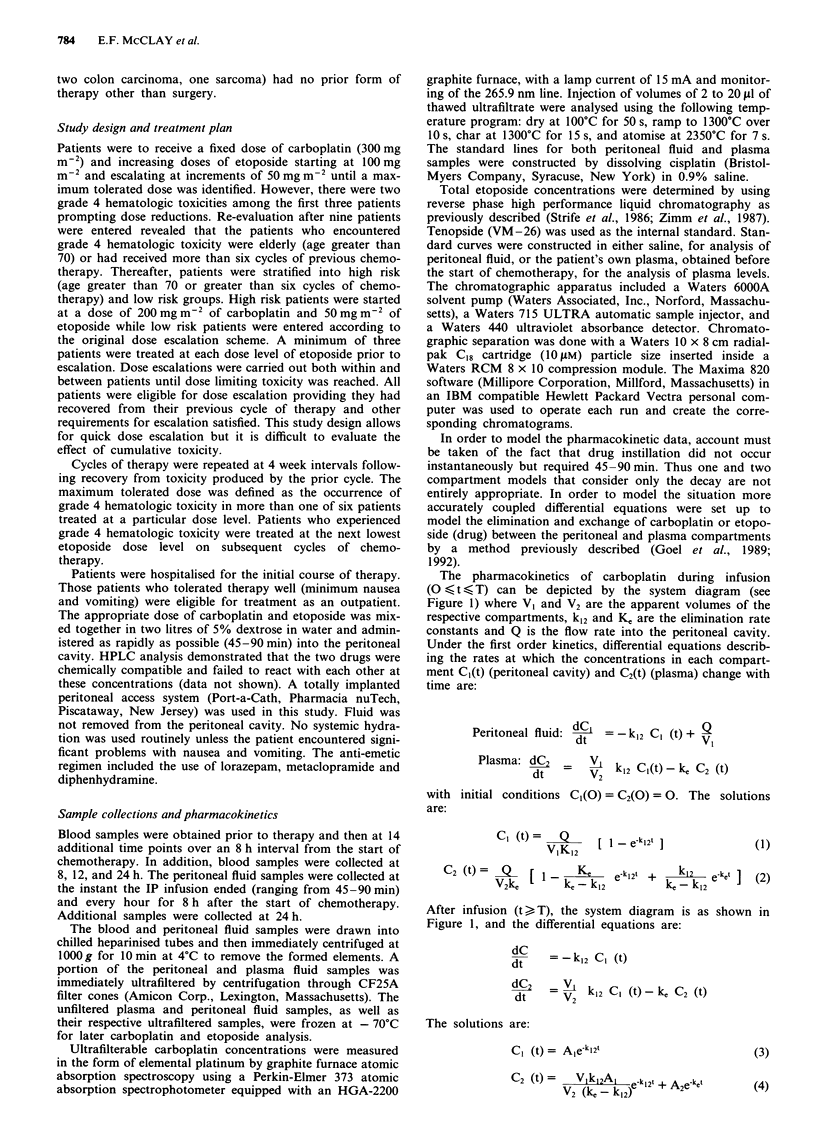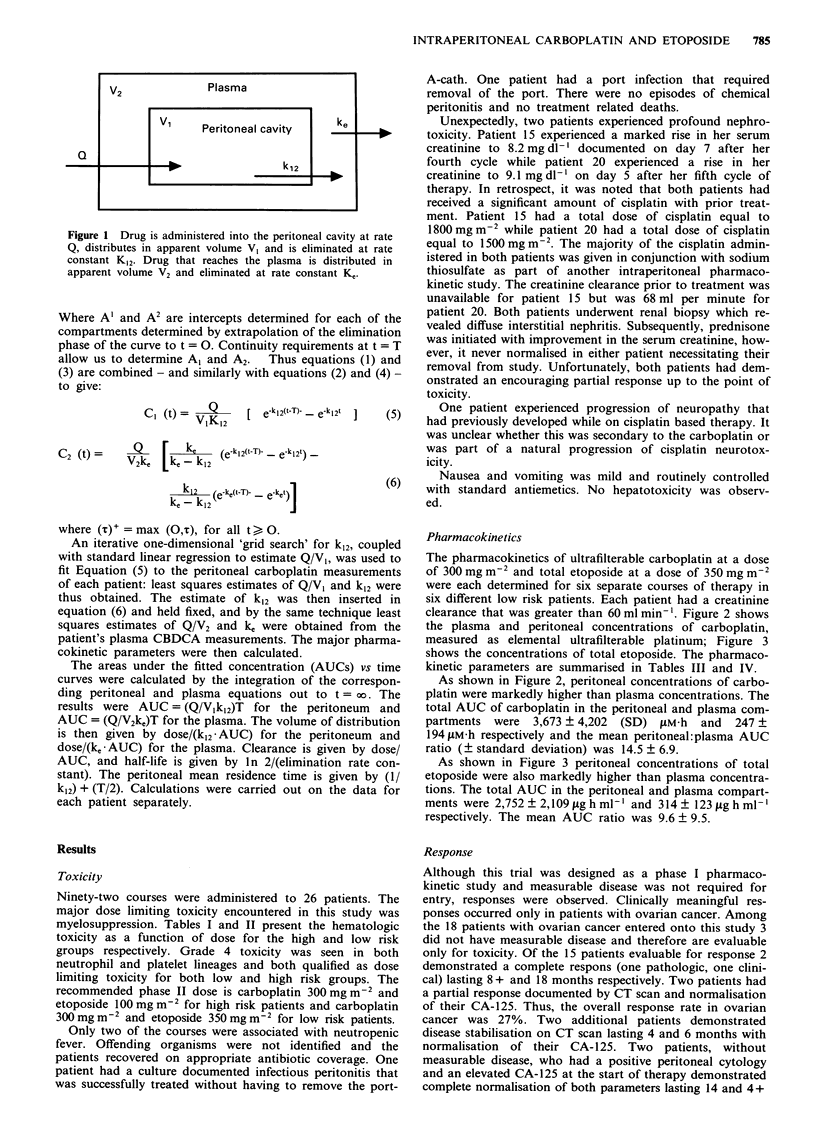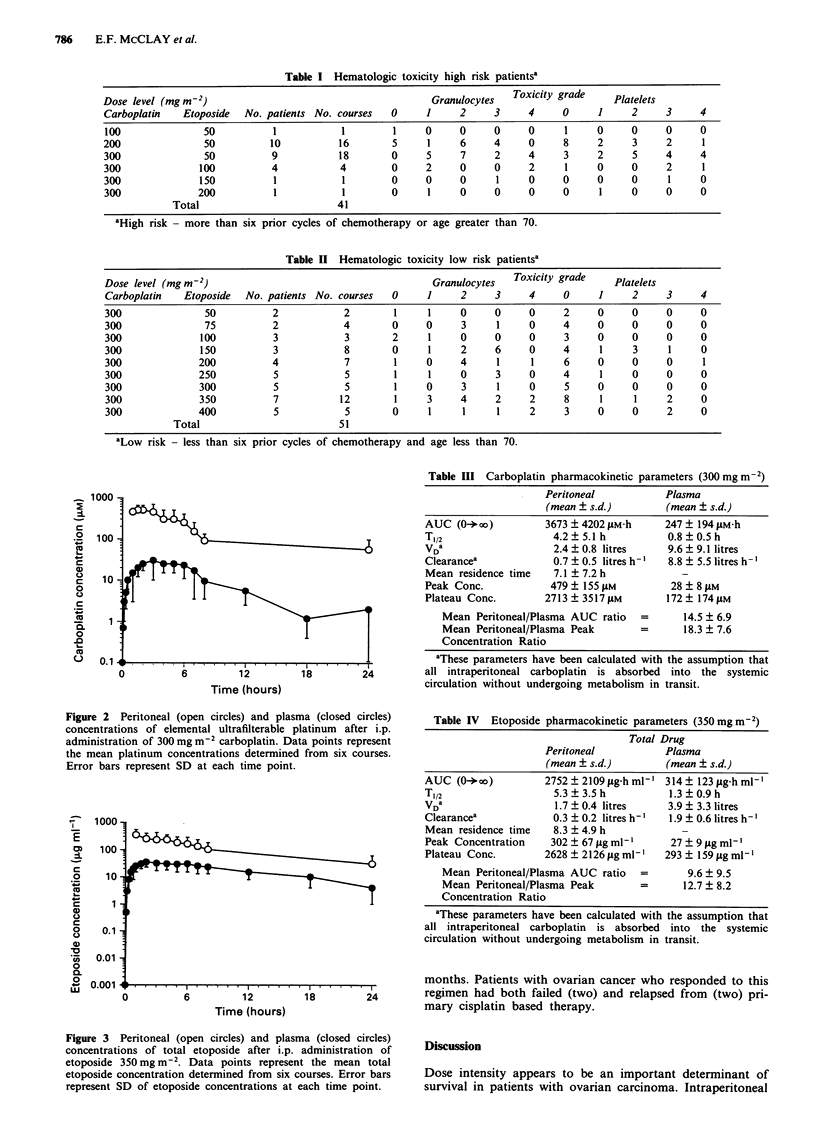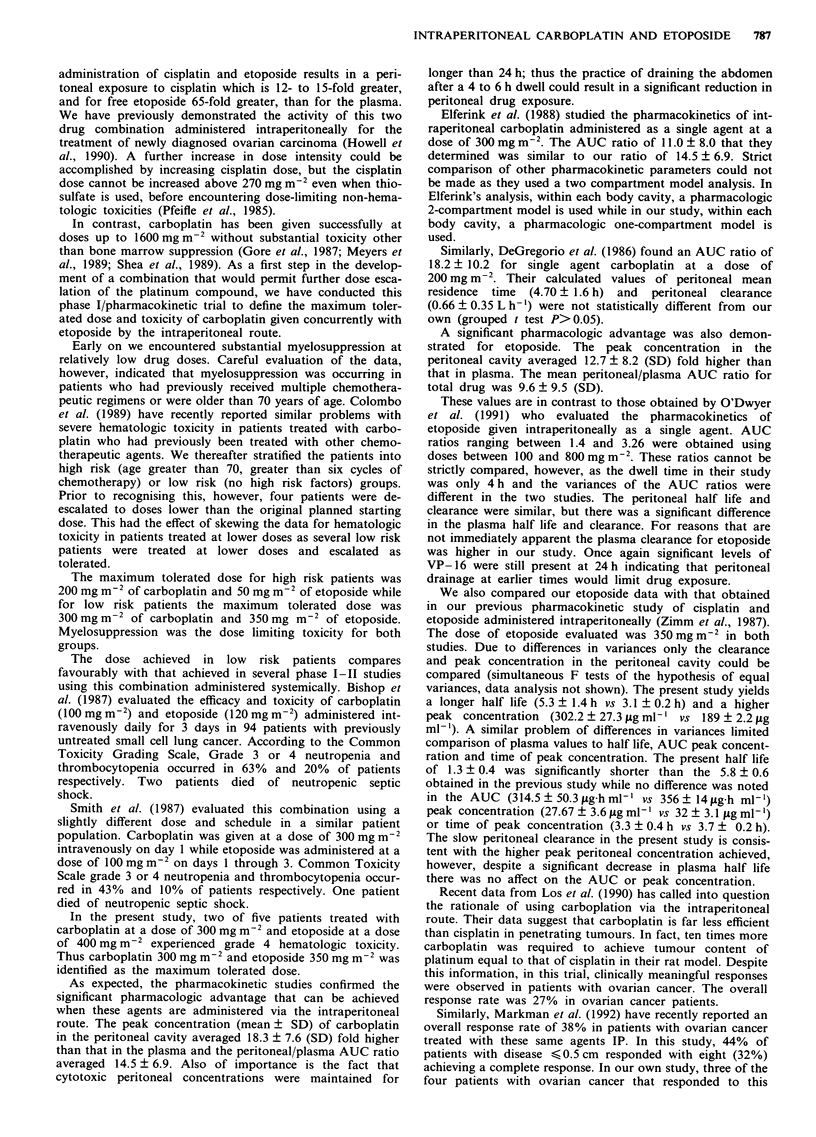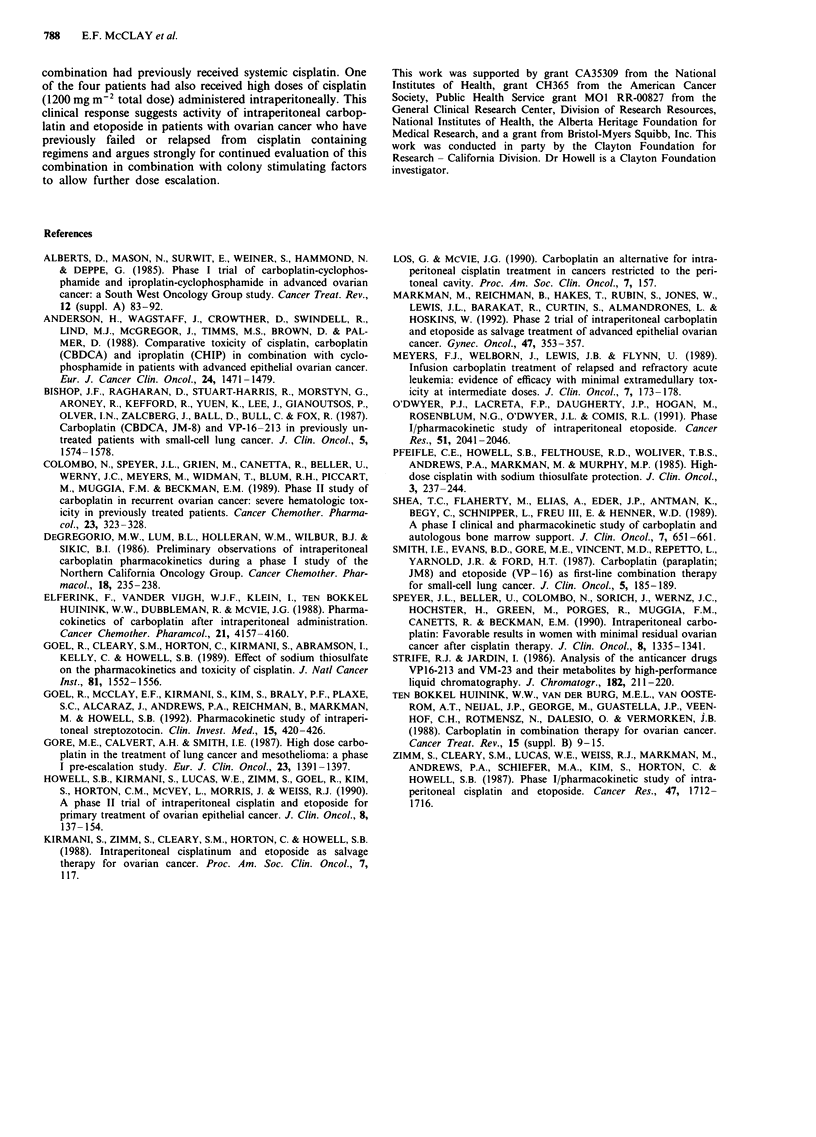# A phase I and pharmacokinetic study of intraperitoneal carboplatin and etoposide.

**DOI:** 10.1038/bjc.1993.428

**Published:** 1993-10

**Authors:** E. F. McClay, R. Goel, P. Andrews, S. Gorelick, S. Kirmani, S. Kim, P. Braly, S. Plaxe, S. Hoff, J. Alcaraz

**Affiliations:** Department of Medicine, University of California, San Diego 92103.

## Abstract

BACKGROUND: We attempted to determine the maximum tolerated dose and toxicity of etoposide (VP-16) when administered in combination with carboplatin (CBDCA) (300 mg m-2) and administered via the intraperitoneal (IP) route. METHODS AND MATERIALS: A total of 26 patients were treated on this trial. CBDCA was administered at a fixed dose of 300 mg m-2) while VP-16 was started at a dose of 200 mg m-2 and escalated at 50 mg m-2 increments. Both agents were mixed together in 2 litres of 5% Dextrose and administered as quickly as possible into the peritoneal cavity. Pharmacokinetic studies were performed at the maximum tolerated dose (MTD). RESULTS: The MTD for this regimen was CBDCA 300 mg m-2 and VP-16 350 mg m-2. Patients > or = 70 years of age or who had received more than six cycles of previous chemotherapy, tolerated this regimen poorly. The MTD for this group of patients was CBDCA 200 mg m-2 and VP-16 50 mg m-2. Neutropenia was the dose limiting toxicity for both groups. The mean peritoneal/plasma peak ratio was 18.3 for CBDCA and 12.7 for VP-16. The pharmacologic advantage (peritoneal/plasma AUC ratio) was 14.9 for CBDCA and 8.8 for VP-16. Although measurable disease was not a requirement for entrance into this study a response rate of 27% was noted in 15 patients with evaluable disease who had ovarian cancer. CONCLUSIONS: A pharmacologic advantage exists for both CBDCA and VP-16 when administered together via the IP route.


*Br. J. Cancer* (1993), **68**, 783–788 © Macmillan Press Ltd., 1993

### A phase I and pharmacokinetic study of intraperitoneal carboplatin and etoposide

E.F. McClay[1], R. Goel[2], P. Andrews[3], S. Gorelick[1], S. Kirmani[1], S. Kim[1], P. Braly[4], S. Plaxe[4], S. Hoff[4], J. Alcaraz[1] & S.B. Howell[1]

[1]*Department of Medicine, University of California, San Diego, UCSD Cancer Center, San Diego, California 92103;* [2]*Civic Division, Ottawa Regional Cancer Center, 190 Melrose, Ottawa, Ontario K1Y 4K7, Canada;* [3]*Department of Pharmacology, Georgetown University, Rockville Maryland 20850;* [4]*Department of Reproductive Medicine, University of California, San Diego, California 92103, USA.*

**Summary** *Background* We attempted to determine the maximum tolerated dose and toxicity of etoposide (VP-16) when administered in combination with carboplatin (CBDCA) (300 mg m$^{-2}$) and administered via the intraperitoneal (IP) route.

*Methods and Materials* A total of 26 patients were treated on this trial. CBDCA was administered at a fixed dose of 300 mg m$^{-2}$) while VP-16 was started at a dose of 200 mg m$^{-2}$ and escalated at 50 mg m$^{-2}$ increments. Both agents were mixed together in 2 litres of 5% Dextrose and administered as quickly as possible into the peritoneal cavity. Pharmacokinetic studies were performed at the maximum tolerated dose (MTD).

*Results* The MTD for this regimen was CBDCA 300 mg m$^{-2}$ and VP-16 350 mg m$^{-2}$. Patients $\geqslant$ 70 years of age or who had received more than six cycles of previous chemotherapy, tolerated this regimen poorly. The MTD for this group of patients was CBDCA 200 mg m$^{-2}$ and VP-16 50 mg m$^{-2}$. Neutropenia was the dose limiting toxicity for both groups. The mean peritoneal/plasma peak ratio was 18.3 for CBDCA and 12.7 for VP-16. The pharmacologic advantage (peritoneal/plasma AUC ratio) was 14.9 for CBDCA and 8.8 for VP-16. Although measurable disease was not a requirement for entrance into this study a response rate of 27% was noted in 15 patients with evaluable disease who had ovarian cancer.

*Conclusions* A pharmacologic advantage exists for both CBDCA and VP-16 when administered together via the IP route.

We have recently completed phase I and II trials of the combination of high dose cisplatin and etoposide administered concurrently via the intraperitoneal route (Howell *et al.*, 1990; Kirmani *et al.*, 1988; Zimm *et al.*, 1987). This combination demonstrated substantial activity as both a salvage regimen as well as initial therapy (Howell *et al.*, 1990; Kirmani *et al.*, 1988). This information has been used to design a phase III prospective randomised trial comparing standard intravenous cisplatin and cyclophosphamide with high dose cisplatin and etoposide administered intraperitoneally that is currently ongoing at the UCSD Cancer Center.

Carboplatin, a cisplatin analogue, has recently been approved for use for the treatment of ovarian cancer by the Food and Drug Administration. It has demonstrated substantially less nephrotoxicity, neurotoxicity and ototoxicity while it appears to be as effective as cisplatin for the treatment of ovarian cancer when used in appropriate doses (Alberts *et al.*, 1985; Anderson *et al.*, 1988; ten Bokkel Huinink *et al.*, 1988). Additionally two phase I and one phase II trials of the intraperitoneal administration of carboplatin have been completed (DeGregorio *et al.*, 1986; Elferink *et al.*, 1988; Speyer *et al.*, 1990). In patients with creatinine clearances of greater than 60 ml per minute the maximum tolerated dose was in excess of 400 mg m$^{-2}$ in both studies. The dose limiting toxicity was myelosuppression with little evidence of chemical peritonitis. Pharmacokinetic studies have demonstrated a peritoneal to plasma AUC ratio of approximately 14-fold. Clinical responses were observed in all of the studies.

Based on the activity of the intraperitoneal cisplatin/etoposide combination and the difference in the pattern of toxicities of carboplatin, we sought to determine whether or not carboplatin could be substituted for cisplatin in this combination. The purposes of this trial were to: (1) determine the maximum tolerated dose of etoposide that can be administered via the IP route concurrently with a fixed dose of carboplatin (300 mg m$^{-2}$), (2) determine the pattern of toxicity produced by this drug combination given by the IP route, (3) determine the intraperitoneal pharmacokinetics of each agent at the recommended phase II dose level.

Correspondence: E.F. McClay, M.D., 225 Dickinson Street, H-811K, San Diego, California 92103, USA.
Received 11 March 1993; and in revised form 1 June 1993.

### Material and methods

### *Patient eligibility and characteristics*

Patients were eligible for participation in this trial if they were 18 years of age or older and had a histologically proven diagnosis of cancer that was refractory to or relapsed after conventional modes of therapy or for which no effective chemotherapy exists. All patients were fully ambulatory and had a life expectancy of at least eight weeks. Patients had to have recovered from the toxic effects of prior therapy and have normal renal (creatinine $\leqslant$ 1.5 mg dl$^{-1}$) and hepatic function (bilirubin <1.5 mg%, SGOT less than three times the upper limit of normal). Hematologic parameters required a granulocyte count greater than 1,500 mm$^{-3}$ and platelet count greater than 100,000 mm$^{-3}$. Written informed consent was obtained prior to entry on the study. The common toxicity scale was used to grade toxicity.

Twenty-six patients were entered. All were evaluable for toxicity. While 21 are evaluable for response on the basis of having either a lesion measurable on physical exam or radiographic study or at subsequent laparotomy. Five patients did not have measurable disease upon entry onto the study. A total of 91 evaluable courses were administered to these patients. Twenty-one patients were female and five were male. The median age was 59 with a range from 26 to 83. ECOG performance status ranged from 0 to 2. Eighteen patients had ovarian cancer, three had gastric, two had colon, and one each had fallopian tube, pancreas and sarcoma. Nineteen patients had received prior chemotherapy, and seven had received prior radiation therapy. Seventeen patients had received prior cisplatin containing regimens, and eight had received prior intraperitoneal chemotherapy. Six patients (two gastric carcinoma, one pancreatic carcinoma, two colon carcinoma, one sarcoma) had no prior form of therapy other than surgery.

*Study design and treatment plan*

Patients were to receive a fixed dose of carboplatin (300 mg $m^{-2}$) and increasing doses of etoposide starting at 100 mg $m^{-2}$ and escalating at increments of 50 mg $m^{-2}$ until a maximum tolerated dose was identified. However, there were two grade 4 hematologic toxicities among the first three patients prompting dose reductions. Re-evaluation after nine patients were entered revealed that the patients who encountered grade 4 hematologic toxicity were elderly (age greater than 70) or had received more than six cycles of previous chemotherapy. Thereafter, patients were stratified into high risk (age greater than 70 or greater than six cycles of chemotherapy) and low risk groups. High risk patients were started at a dose of 200 mg $m^{-2}$ of carboplatin and 50 mg $m^{-2}$ of etoposide while low risk patients were entered according to the original dose escalation scheme. A minimum of three patients were treated at each dose level of etoposide prior to escalation. Dose escalations were carried out both within and between patients until dose limiting toxicity was reached. All patients were eligible for dose escalation providing they had recovered from their previous cycle of therapy and other requirements for escalation satisfied. This study design allows for quick dose escalation but it is difficult to evaluate the effect of cumulative toxicity.

Cycles of therapy were repeated at 4 week intervals following recovery from toxicity produced by the prior cycle. The maximum tolerated dose was defined as the occurrence of grade 4 hematologic toxicity in more than one of six patients treated at a particular dose level. Patients who experienced grade 4 hematologic toxicity were treated at the next lowest etoposide dose level on subsequent cycles of chemotherapy.

Patients were hospitalised for the initial course of therapy. Those patients who tolerated therapy well (minimum nausea and vomiting) were eligible for treatment as an outpatient. The appropriate dose of carboplatin and etoposide was mixed together in two litres of 5% dextrose in water and administered as rapidly as possible (45–90 min) into the peritoneal cavity. HPLC analysis demonstrated that the two drugs were chemically compatible and failed to react with each other at these concentrations (data not shown). A totally implanted peritoneal access system (Port-a-Cath, Pharmacia nuTech, Piscataway, New Jersey) was used in this study. Fluid was not removed from the peritoneal cavity. No systemic hydration was used routinely unless the patient encountered significant problems with nausea and vomiting. The anti-emetic regimen included the use of lorazepam, metaclopramide and diphenhydramine.

*Sample collections and pharmacokinetics*

Blood samples were obtained prior to therapy and then at 14 additional time points over an 8 h interval from the start of chemotherapy. In addition, blood samples were collected at 8, 12, and 24 h. The peritoneal fluid samples were collected at the instant the IP infusion ended (ranging from 45–90 min) and every hour for 8 h after the start of chemotherapy. Additional samples were collected at 24 h.

The blood and peritoneal fluid samples were drawn into chilled heparinised tubes and then immediately centrifuged at 1000 ***g*** for 10 min at 4°C to remove the formed elements. A portion of the peritoneal and plasma fluid samples was immediately ultrafiltered by centrifugation through CF25A filter cones (Amicon Corp., Lexington, Massachusetts). The unfiltered plasma and peritoneal fluid samples, as well as their respective ultrafiltered samples, were frozen at − 70°C for later carboplatin and etoposide analysis.

Ultrafilterable carboplatin concentrations were measured in the form of elemental platinum by graphite furnace atomic absorption spectroscopy using a Perkin-Elmer 373 atomic absorption spectrophotometer equipped with an HGA-2200 graphite furnace, with a lamp current of 15 mA and monitoring of the 265.9 nm line. Injection of volumes of 2 to 20 µl of thawed ultrafiltrate were analysed using the following temperature program: dry at 100°C for 50 s, ramp to 1300°C over 10 s, char at 1300°C for 15 s, and atomise at 2350°C for 7 s. The standard lines for both peritoneal fluid and plasma samples were constructed by dissolving cisplatin (Bristol-Myers Company, Syracuse, New York) in 0.9% saline.

Total etoposide concentrations were determined by using reverse phase high performance liquid chromatography as previously described (Strife *et al.*, 1986; Zimm *et al.*, 1987). Tenopside (VM−26) was used as the internal standard. Standard curves were constructed in either saline, for analysis of peritoneal fluid, or the patient's own plasma, obtained before the start of chemotherapy, for the analysis of plasma levels. The chromatographic apparatus included a Waters 6000A solvent pump (Waters Associated, Inc., Norford, Massachusetts), a Waters 715 ULTRA automatic sample injector, and a Waters 440 ultraviolet absorbance detector. Chromatographic separation was done with a Waters 10 × 8 cm radial-pak $C_{18}$ cartridge (10 µM) particle size inserted inside a Waters RCM 8 × 10 compression module. The Maxima 820 software (Millipore Corporation, Millford, Massachusetts) in an IBM compatible Hewlett Packard Vectra personal computer was used to operate each run and create the corresponding chromatograms.

In order to model the pharmacokinetic data, account must be taken of the fact that drug instillation did not occur instantaneously but required 45–90 min. Thus one and two compartment models that consider only the decay are not entirely appropriate. In order to model the situation more accurately coupled differential equations were set up to model the elimination and exchange of carboplatin or etoposide (drug) between the peritoneal and plasma compartments by a method previously described (Goel *et al.*, 1989; 1992).

The pharmacokinetics of carboplatin during infusion ($O \leqslant t \leqslant T$) can be depicted by the system diagram (see Figure 1) where $V_1$ and $V_2$ are the apparent volumes of the respective compartments, $k_{12}$ and $K_e$ are the elimination rate constants and Q is the flow rate into the peritoneal cavity. Under the first order kinetics, differential equations describing the rates at which the concentrations in each compartment $C_1(t)$ (peritoneal cavity) and $C_2(t)$ (plasma) change with time are:

$$\text{Peritoneal fluid: } \frac{dC_1}{dt} = -k_{12}\, C_1\,(t) + \frac{Q}{V_1}$$

$$\text{Plasma: } \frac{dC_2}{dt} = \frac{V_1}{V_2}\, k_{12}\, C_1(t) - k_e\, C_2\,(t)$$

with initial conditions $C_1(O) = C_2(O) = O$. The solutions are:

$$C_1\,(t) = \frac{Q}{V_1 K_{12}}\,[\,1 - e^{-k_{12}t}\,] \quad (1)$$

$$C_2\,(t) = \frac{Q}{V_2 k_e}\,[\,1 - \frac{K_e}{k_e - k_{12}}\,e^{-k_{12}t} + \frac{k_{12}}{k_e - k_{12}}\,e^{-k_e t}\,] \quad (2)$$

After infusion ($t \geqslant T$), the system diagram is as shown in Figure 1, and the differential equations are:

$$\frac{dC}{dt} = -k_{12}\, C_1\,(t)$$

$$\frac{dC_2}{dt} = \frac{V_1}{V_2}\, k_{12}\, C_1\,(t) - k_e\, C_2\,(t)$$

The solutions are:

$$C_1\,(t) = A_1 e^{-k_{12}t} \quad (3)$$

$$C_2\,(t) = \frac{V_1 k_{12} A_1}{V_2\,(k_e - k_{12})} e^{-k_{12}t} + A_2 e^{-k_e t} \quad (4)$$

**Figure 1** Drug is administered into the peritoneal cavity at rate Q, distributes in apparent volume $V_1$ and is eliminated at rate constant $K_{12}$. Drug that reaches the plasma is distributed in apparent volume $V_2$ and eliminated at rate constant $K_e$.

Where $A^1$ and $A^2$ are intercepts determined for each of the compartments determined by extrapolation of the elimination phase of the curve to t = O. Continuity requirements at t = T allow us to determine $A_1$ and $A_2$. Thus equations (1) and (3) are combined – and similarly with equations (2) and (4) – to give:

$$C_1\ (t) = \frac{Q}{V_1K_{12}}\ [\ e^{-k_{12}(t-T)^{+}} - e^{-k_{12}t}\ ] \qquad (5)$$

$$C_2\ (t) = \frac{Q}{V_2k_e}\left[\frac{k_e}{k_e - k_{12}}\left(e^{-k_{12}(t-T)^{+}} - e^{-k_{12}t}\right) - \frac{k_{12}}{k_e - k_{12}}\left(e^{-k_e(t-T)^{+}} - e^{-k_et}\right)\right] \qquad (6)$$

where $(\tau)^+ = \max\ (O,\tau)$, for all $t \geqslant O$.

An iterative one-dimensional 'grid search' for $k_{12}$, coupled with standard linear regression to estimate $Q/V_1$, was used to fit Equation (5) to the peritoneal carboplatin measurements of each patient: least squares estimates of $Q/V_1$ and $k_{12}$ were thus obtained. The estimate of $k_{12}$ was then inserted in equation (6) and held fixed, and by the same technique least squares estimates of $Q/V_2$ and $k_e$ were obtained from the patient's plasma CBDCA measurements. The major pharmacokinetic parameters were then calculated.

The areas under the fitted concentration (AUCs) *vs* time curves were calculated by the integration of the corresponding peritoneal and plasma equations out to $t = \infty$. The results were $AUC = (Q/V_1k_{12})T$ for the peritoneum and $AUC = (Q/V_2k_e)T$ for the plasma. The volume of distribution is then given by dose/($k_{12}\cdot$AUC) for the peritoneum and dose/($k_e\cdot$AUC) for the plasma. Clearance is given by dose/AUC, and half-life is given by ln 2/(elimination rate constant). The peritoneal mean residence time is given by ($1/k_{12}$) + (T/2). Calculations were carried out on the data for each patient separately.

### Results

### *Toxicity*

Ninety-two courses were administered to 26 patients. The major dose limiting toxicity encountered in this study was myelosuppression. Tables I and II present the hematologic toxicity as a function of dose for the high and low risk groups respectively. Grade 4 toxicity was seen in both neutrophil and platelet lineages and both qualified as dose limiting toxicity for both low and high risk groups. The recommended phase II dose is carboplatin 300 mg $m^{-2}$ and etoposide 100 mg $m^{-2}$ for high risk patients and carboplatin 300 mg $m^{-2}$ and etoposide 350 mg $m^{-2}$ for low risk patients.

Only two of the courses were associated with neutropenic fever. Offending organisms were not identified and the patients recovered on appropriate antibiotic coverage. One patient had a culture documented infectious peritonitis that was successfully treated without having to remove the port-A-cath. One patient had a port infection that required removal of the port. There were no episodes of chemical peritonitis and no treatment related deaths.

Unexpectedly, two patients experienced profound nephrotoxicity. Patient 15 experienced a marked rise in her serum creatinine to 8.2 mg $dl^{-1}$ documented on day 7 after her fourth cycle while patient 20 experienced a rise in her creatinine to 9.1 mg $dl^{-1}$ on day 5 after her fifth cycle of therapy. In retrospect, it was noted that both patients had received a significant amount of cisplatin with prior treatment. Patient 15 had a total dose of cisplatin equal to 1800 mg $m^{-2}$ while patient 20 had a total dose of cisplatin equal to 1500 mg $m^{-2}$. The majority of the cisplatin administered in both patients was given in conjunction with sodium thiosulfate as part of another intraperitoneal pharmacokinetic study. The creatinine clearance prior to treatment was unavailable for patient 15 but was 68 ml per minute for patient 20. Both patients underwent renal biopsy which revealed diffuse interstitial nephritis. Subsequently, prednisone was initiated with improvement in the serum creatinine, however, it never normalised in either patient necessitating their removal from study. Unfortunately, both patients had demonstrated an encouraging partial response up to the point of toxicity.

One patient experienced progression of neuropathy that had previously developed while on cisplatin based therapy. It was unclear whether this was secondary to the carboplatin or was part of a natural progression of cisplatin neurotoxicity.

Nausea and vomiting was mild and routinely controlled with standard antiemetics. No hepatotoxicity was observed.

### *Pharmacokinetics*

The pharmacokinetics of ultrafilterable carboplatin at a dose of 300 mg $m^{-2}$ and total etoposide at a dose of 350 mg $m^{-2}$ were each determined for six separate courses of therapy in six different low risk patients. Each patient had a creatinine clearance that was greater than 60 ml $min^{-1}$. Figure 2 shows the plasma and peritoneal concentrations of carboplatin, measured as elemental ultrafilterable platinum; Figure 3 shows the concentrations of total etoposide. The pharmacokinetic parameters are summarised in Tables III and IV.

As shown in Figure 2, peritoneal concentrations of carboplatin were markedly higher than plasma concentrations. The total AUC of carboplatin in the peritoneal and plasma compartments were 3,673 ± 4,202 (SD) μM·h and 247 ± 194 μM·h respectively and the mean peritoneal:plasma AUC ratio (± standard deviation) was 14.5 ± 6.9.

As shown in Figure 3 peritoneal concentrations of total etoposide were also markedly higher than plasma concentrations. The total AUC in the peritoneal and plasma compartments were 2,752 ± 2,109 μg h $ml^{-1}$ and 314 ± 123 μg h $ml^{-1}$ respectively. The mean AUC ratio was 9.6 ± 9.5.

### *Response*

Although this trial was designed as a phase I pharmacokinetic study and measurable disease was not required for entry, responses were observed. Clinically meaningful responses occurred only in patients with ovarian cancer. Among the 18 patients with ovarian cancer entered onto this study 3 did not have measurable disease and therefore are evaluable only for toxicity. Of the 15 patients evaluable for response 2 demonstrated a complete respons (one pathologic, one clinical) lasting 8+ and 18 months respectively. Two patients had a partial response documented by CT scan and normalisation of their CA-125. Thus, the overall response rate in ovarian cancer was 27%. Two additional patients demonstrated disease stabilisation on CT scan lasting 4 and 6 months with normalisation of their CA-125. Two patients, without measurable disease, who had a positive peritoneal cytology and an elevated CA-125 at the start of therapy demonstrated complete normalisation of both parameters lasting 14 and 4+

**Table I** Hematologic toxicity high risk patients[a]

| Dose level ($mg\,m^{-2}$) Carboplatin | Etoposide | No. patients | No. courses | Toxicity grade: Granulocytes 0 | 1 | 2 | 3 | 4 | Platelets 0 | 1 | 2 | 3 | 4 |
|---|---|---|---|---|---|---|---|---|---|---|---|---|---|
| 100 | 50 | 1 | 1 | 1 | 0 | 0 | 0 | 0 | 1 | 0 | 0 | 0 | 0 |
| 200 | 50 | 10 | 16 | 5 | 1 | 6 | 4 | 0 | 8 | 2 | 3 | 2 | 1 |
| 300 | 50 | 9 | 18 | 0 | 5 | 7 | 2 | 4 | 3 | 2 | 5 | 4 | 4 |
| 300 | 100 | 4 | 4 | 0 | 2 | 0 | 0 | 2 | 1 | 0 | 0 | 2 | 1 |
| 300 | 150 | 1 | 1 | 0 | 0 | 0 | 1 | 0 | 0 | 0 | 0 | 1 | 0 |
| 300 | 200 | 1 | 1 | 0 | 1 | 0 | 0 | 0 | 0 | 1 | 0 | 0 | 0 |
| Total | | | 41 | | | | | | | | | | |

[a]High risk – more than six prior cycles of chemotherapy or age greater than 70.

**Table II** Hematologic toxicity low risk patients[a]

| Dose level ($mg\,m^{-2}$) Carboplatin | Etoposide | No. patients | No. courses | Toxicity grade: Granulocytes 0 | 1 | 2 | 3 | 4 | Platelets 0 | 1 | 2 | 3 | 4 |
|---|---|---|---|---|---|---|---|---|---|---|---|---|---|
| 300 | 50 | 2 | 2 | 1 | 1 | 0 | 0 | 0 | 2 | 0 | 0 | 0 | 0 |
| 300 | 75 | 2 | 4 | 0 | 0 | 3 | 1 | 0 | 4 | 0 | 0 | 0 | 0 |
| 300 | 100 | 3 | 3 | 2 | 1 | 0 | 0 | 0 | 3 | 0 | 0 | 0 | 0 |
| 300 | 150 | 3 | 8 | 0 | 1 | 2 | 6 | 0 | 4 | 1 | 3 | 1 | 0 |
| 300 | 200 | 4 | 7 | 1 | 0 | 4 | 1 | 1 | 6 | 0 | 0 | 0 | 1 |
| 300 | 250 | 5 | 5 | 1 | 1 | 0 | 3 | 0 | 4 | 1 | 0 | 0 | 0 |
| 300 | 300 | 5 | 5 | 1 | 0 | 3 | 1 | 0 | 5 | 0 | 0 | 0 | 0 |
| 300 | 350 | 7 | 12 | 1 | 3 | 4 | 2 | 2 | 8 | 1 | 1 | 2 | 0 |
| 300 | 400 | 5 | 5 | 0 | 1 | 1 | 1 | 2 | 3 | 0 | 0 | 2 | 0 |
| Total | | | 51 | | | | | | | | | | |

[a]Low risk – less than six prior cycles of chemotherapy and age less than 70.

**Figure 2** Peritoneal (open circles) and plasma (closed circles) concentrations of elemental ultrafilterable platinum after i.p. administration of 300 mg m$^{-2}$ carboplatin. Data points represent the mean platinum concentrations determined from six courses. Error bars represent SD at each time point.

**Figure 3** Peritoneal (open circles) and plasma (closed circles) concentrations of total etoposide after i.p. administration of etoposide 350 mg m$^{-2}$. Data points represent the mean total etoposide concentration determined from six courses. Error bars represent SD of etoposide concentrations at each time point.

**Table III** Carboplatin pharmacokinetic parameters (300 mg m$^{-2}$)

| | Peritoneal (mean ± s.d.) | Plasma (mean ± s.d.) |
|---|---|---|
| AUC (0→∞) | 3673 ± 4202 μM·h | 247 ± 194 μM·h |
| $T_{1/2}$ | 4.2 ± 5.1 h | 0.8 ± 0.5 h |
| $V_D$[a] | 2.4 ± 0.8 litres | 9.6 ± 9.1 litres |
| Clearance[a] | 0.7 ± 0.5 litres h$^{-1}$ | 8.8 ± 5.5 litres h$^{-1}$ |
| Mean residence time | 7.1 ± 7.2 h | – |
| Peak Conc. | 479 ± 155 μM | 28 ± 8 μM |
| Plateau Conc. | 2713 ± 3517 μM | 172 ± 174 μM |
| Mean Peritoneal/Plasma AUC ratio | = | 14.5 ± 6.9 |
| Mean Peritoneal/Plasma Peak Concentration Ratio | = | 18.3 ± 7.6 |

[a]These parameters have been calculated with the assumption that all intraperitoneal carboplatin is absorbed into the systemic circulation without undergoing metabolism in transit.

**Table IV** Etoposide pharmacokinetic parameters (350 mg m$^{-2}$)

| | Total Drug Peritoneal (mean ± s.d.) | Plasma (mean ± s.d.) |
|---|---|---|
| AUC (0→∞) | 2752 ± 2109 μg·h ml$^{-1}$ | 314 ± 123 μg·h ml$^{-1}$ |
| $T_{1/2}$ | 5.3 ± 3.5 h | 1.3 ± 0.9 h |
| $V_D$[a] | 1.7 ± 0.4 litres | 3.9 ± 3.3 litres |
| Clearance[a] | 0.3 ± 0.2 litres h$^{-1}$ | 1.9 ± 0.6 litres h$^{-1}$ |
| Mean residence time | 8.3 ± 4.9 h | – |
| Peak Concentration | 302 ± 67 μg ml$^{-1}$ | 27 ± 9 μg ml$^{-1}$ |
| Plateau Conc. | 2628 ± 2126 μg ml$^{-1}$ | 293 ± 159 μg ml$^{-1}$ |
| Mean Peritoneal/Plasma AUC ratio | = | 9.6 ± 9.5 |
| Mean Peritoneal/Plasma Peak Concentration Ratio | = | 12.7 ± 8.2 |

[a]These parameters have been calculated with the assumption that all intraperitoneal carboplatin is absorbed into the systemic circulation without undergoing metabolism in transit.

months. Patients with ovarian cancer who responded to this regimen had both failed (two) and relapsed from (two) primary cisplatin based therapy.

### Discussion

Dose intensity appears to be an important determinant of survival in patients with ovarian carcinoma. Intraperitoneal administration of cisplatin and etoposide results in a peritoneal exposure to cisplatin which is 12- to 15-fold greater, and for free etoposide 65-fold greater, than for the plasma. We have previously demonstrated the activity of this two drug combination administered intraperitoneally for the treatment of newly diagnosed ovarian carcinoma (Howell *et al.*, 1990). A further increase in dose intensity could be accomplished by increasing cisplatin dose, but the cisplatin dose cannot be increased above 270 mg $m^{-2}$ even when thiosulfate is used, before encountering dose-limiting non-hematologic toxicities (Pfeifle *et al.*, 1985).

In contrast, carboplatin has been given successfully at doses up to 1600 mg $m^{-2}$ without substantial toxicity other than bone marrow suppression (Gore *et al.*, 1987; Meyers *et al.*, 1989; Shea *et al.*, 1989). As a first step in the development of a combination that would permit further dose escalation of the platinum compound, we have conducted this phase I/pharmacokinetic trial to define the maximum tolerated dose and toxicity of carboplatin given concurrently with etoposide by the intraperitoneal route.

Early on we encountered substantial myelosuppression at relatively low drug doses. Careful evaluation of the data, however, indicated that myelosuppression was occurring in patients who had previously received multiple chemotherapeutic regimens or were older than 70 years of age. Colombo *et al.* (1989) have recently reported similar problems with severe hematologic toxicity in patients treated with carboplatin who had previously been treated with other chemotherapeutic agents. We thereafter stratified the patients into high risk (age greater than 70, greater than six cycles of chemotherapy) or low risk (no high risk factors) groups. Prior to recognising this, however, four patients were de-escalated to doses lower than the original planned starting dose. This had the effect of skewing the data for hematologic toxicity in patients treated at lower doses as several low risk patients were treated at lower doses and escalated as tolerated.

The maximum tolerated dose for high risk patients was 200 mg $m^{-2}$ of carboplatin and 50 mg $m^{-2}$ of etoposide while for low risk patients the maximum tolerated dose was 300 mg $m^{-2}$ of carboplatin and 350 mg $m^{-2}$ of etoposide. Myelosuppression was the dose limiting toxicity for both groups.

The dose achieved in low risk patients compares favourably with that achieved in several phase I–II studies using this combination administered systemically. Bishop *et al.* (1987) evaluated the efficacy and toxicity of carboplatin (100 mg $m^{-2}$) and etoposide (120 mg $m^{-2}$) administered intravenously daily for 3 days in 94 patients with previously untreated small cell lung cancer. According to the Common Toxicity Grading Scale, Grade 3 or 4 neutropenia and thrombocytopenia occurred in 63% and 20% of patients respectively. Two patients died of neutropenic septic shock.

Smith *et al.* (1987) evaluated this combination using a slightly different dose and schedule in a similar patient population. Carboplatin was given at a dose of 300 mg $m^{-2}$ intravenously on day 1 while etoposide was administered at a dose of 100 mg $m^{-2}$ on days 1 through 3. Common Toxicity Scale grade 3 or 4 neutropenia and thrombocytopenia occurred in 43% and 10% of patients respectively. One patient died of neutropenic septic shock.

In the present study, two of five patients treated with carboplatin at a dose of 300 mg $m^{-2}$ and etoposide at a dose of 400 mg $m^{-2}$ experienced grade 4 hematologic toxicity. Thus carboplatin 300 mg $m^{-2}$ and etoposide 350 mg $m^{-2}$ was identified as the maximum tolerated dose.

As expected, the pharmacokinetic studies confirmed the significant pharmacologic advantage that can be achieved when these agents are administered via the intraperitoneal route. The peak concentration (mean ± SD) of carboplatin in the peritoneal cavity averaged 18.3 ± 7.6 (SD) fold higher than that in the plasma and the peritoneal/plasma AUC ratio averaged 14.5 ± 6.9. Also of importance is the fact that cytotoxic peritoneal concentrations were maintained for longer than 24 h; thus the practice of draining the abdomen after a 4 to 6 h dwell could result in a significant reduction in peritoneal drug exposure.

Elferink *et al.* (1988) studied the pharmacokinetics of intraperitoneal carboplatin administered as a single agent at a dose of 300 mg $m^{-2}$. The AUC ratio of 11.0 ± 8.0 that they determined was similar to our ratio of 14.5 ± 6.9. Strict comparison of other pharmacokinetic parameters could not be made as they used a two compartment model analysis. In Elferink's analysis, within each body cavity, a pharmacologic 2-compartment model is used while in our study, within each body cavity, a pharmacologic one-compartment model is used.

Similarly, DeGregorio *et al.* (1986) found an AUC ratio of 18.2 ± 10.2 for single agent carboplatin at a dose of 200 mg $m^{-2}$. Their calculated values of peritoneal mean residence time (4.70 ± 1.6 h) and peritoneal clearance (0.66 ± 0.35 L $h^{-1}$) were not statistically different from our own (grouped *t* test $P > 0.05$).

A significant pharmacologic advantage was also demonstrated for etoposide. The peak concentration in the peritoneal cavity averaged 12.7 ± 8.2 (SD) fold higher than that in plasma. The mean peritoneal/plasma AUC ratio for total drug was 9.6 ± 9.5 (SD).

These values are in contrast to those obtained by O'Dwyer *et al.* (1991) who evaluated the pharmacokinetics of etoposide given intraperitoneally as a single agent. AUC ratios ranging between 1.4 and 3.26 were obtained using doses between 100 and 800 mg $m^{-2}$. These ratios cannot be strictly compared, however, as the dwell time in their study was only 4 h and the variances of the AUC ratios were different in the two studies. The peritoneal half life and clearance were similar, but there was a significant difference in the plasma half life and clearance. For reasons that are not immediately apparent the plasma clearance for etoposide was higher in our study. Once again significant levels of VP–16 were still present at 24 h indicating that peritoneal drainage at earlier times would limit drug exposure.

We also compared our etoposide data with that obtained in our previous pharmacokinetic study of cisplatin and etoposide administered intraperitoneally (Zimm *et al.*, 1987). The dose of etoposide evaluated was 350 mg $m^{-2}$ in both studies. Due to differences in variances only the clearance and peak concentration in the peritoneal cavity could be compared (simultaneous F tests of the hypothesis of equal variances, data analysis not shown). The present study yields a longer half life (5.3 ± 1.4 h *vs* 3.1 ± 0.2 h) and a higher peak concentration (302.2 ± 27.3 μg $ml^{-1}$ *vs* 189 ± 2.2 μg $ml^{-1}$). A similar problem of differences in variances limited comparison of plasma values to half life, AUC peak concentration and time of peak concentration. The present half life of 1.3 ± 0.4 was significantly shorter than the 5.8 ± 0.6 obtained in the previous study while no difference was noted in the AUC (314.5 ± 50.3 μg·h $ml^{-1}$ *vs* 356 ± 14 μg·h $ml^{-1}$) peak concentration (27.67 ± 3.6 μg $ml^{-1}$ *vs* 32 ± 3.1 μg $ml^{-1}$) or time of peak concentration (3.3 ± 0.4 h *vs* 3.7 ± 0.2 h). The slow peritoneal clearance in the present study is consistent with the higher peak peritoneal concentration achieved, however, despite a significant decrease in plasma half life there was no affect on the AUC or peak concentration.

Recent data from Los *et al.* (1990) has called into question the rationale of using carboplation via the intraperitoneal route. Their data suggest that carboplatin is far less efficient than cisplatin in penetrating tumours. In fact, ten times more carboplatin was required to achieve tumour content of platinum equal to that of cisplatin in their rat model. Despite this information, in this trial, clinically meaningful responses were observed in patients with ovarian cancer. The overall response rate was 27% in ovarian cancer patients.

Similarly, Markman *et al.* (1992) have recently reported an overall response rate of 38% in patients with ovarian cancer treated with these same agents IP. In this study, 44% of patients with disease ⩽0.5 cm responded with eight (32%) achieving a complete response. In our own study, three of the four patients with ovarian cancer that responded to this combination had previously received systemic cisplatin. One of the four patients had also received high doses of cisplatin ($1200 \text{ mg m}^{-2}$ total dose) administered intraperitoneally. This clinical response suggests activity of intraperitoneal carboplatin and etoposide in patients with ovarian cancer who have previously failed or relapsed from cisplatin containing regimens and argues strongly for continued evaluation of this combination in combination with colony stimulating factors to allow further dose escalation.

This work was supported by grant CA35309 from the National Institutes of Health, grant CH365 from the American Cancer Society, Public Health Service grant MO1 RR-00827 from the General Clinical Research Center, Division of Research Resources, National Institutes of Health, the Alberta Heritage Foundation for Medical Research, and a grant from Bristol-Myers Squibb, Inc. This work was conducted in party by the Clayton Foundation for Research – California Division. Dr Howell is a Clayton Foundation investigator.

### References

ALBERTS, D., MASON, N., SURWIT, E., WEINER, S., HAMMOND, N. & DEPPE, G. (1985). Phase I trial of carboplatin-cyclophosphamide and iproplatin-cyclophosphamide in advanced ovarian cancer: a South West Oncology Group study. *Cancer Treat. Rev.*, **12** (suppl. A) 83–92.

ANDERSON, H., WAGSTAFF, J., CROWTHER, D., SWINDELL, R., LIND, M.J., MCGREGOR, J., TIMMS, M.S., BROWN, D. & PALMER, D. (1988). Comparative toxicity of cisplatin, carboplatin (CBDCA) and iproplatin (CHIP) in combination with cyclophosphamide in patients with advanced epithelial ovarian cancer. *Eur. J. Cancer Clin. Oncol.*, **24**, 1471–1479.

BISHOP, J.F., RAGHARAN, D., STUART-HARRIS, R., MORSTYN, G., ARONEY, R., KEFFORD, R., YUEN, K., LEE, J., GIANOUTSOS, P., OLVER, I.N., ZALCBERG, J., BALL, D., BULL, C. & FOX, R. (1987). Carboplatin (CBDCA, JM-8) and VP-16-213 in previously untreated patients with small-cell lung cancer. *J. Clin. Oncol.*, **5**, 1574–1578.

COLOMBO, N., SPEYER, J.L., GRIEN, M., CANETTA, R., BELLER, U., WERNY, J.C., MEYERS, M., WIDMAN, T., BLUM, R.H., PICCART, M., MUGGIA, F.M. & BECKMAN, E.M. (1989). Phase II study of carboplatin in recurrent ovarian cancer: severe hematologic toxicity in previously treated patients. *Cancer Chemother. Pharmacol.*, **23**, 323–328.

DEGREGORIO, M.W., LUM, B.L., HOLLERAN, W.M., WILBUR, B.J. & SIKIC, B.I. (1986). Preliminary observations of intraperitoneal carboplatin pharmacokinetics during a phase I study of the Northern California Oncology Group. *Cancer Chemother. Pharmacol.*, **18**, 235–238.

ELFERINK, F., VANDER VIJGH, W.J.F., KLEIN, I., TEN BOKKEL HUININK, W.W., DUBBLEMAN, R. & MCVIE, J.G. (1988). Pharmacokinetics of carboplatin after intraperitoneal administration. *Cancer Chemother. Pharamcol.*, **21**, 4157–4160.

GOEL, R., CLEARY, S.M., HORTON, C., KIRMANI, S., ABRAMSON, I., KELLY, C. & HOWELL, S.B. (1989). Effect of sodium thiosulfate on the pharmacokinetics and toxicity of cisplatin. *J. Natl Cancer Inst.*, **81**, 1552–1556.

GOEL, R., MCCLAY, E.F., KIRMANI, S., KIM, S., BRALY, P.F., PLAXE, S.C., ALCARAZ, J., ANDREWS, P.A., REICHMAN, B., MARKMAN, M. & HOWELL, S.B. (1992). Pharmacokinetic study of intraperitoneal streptozotocin. *Clin. Invest. Med.*, **15**, 420–426.

GORE, M.E., CALVERT, A.H. & SMITH, I.E. (1987). High dose carboplatin in the treatment of lung cancer and mesothelioma: a phase I pre-escalation study. *Eur. J. Clin. Oncol.*, **23**, 1391–1397.

HOWELL, S.B., KIRMANI, S., LUCAS, W.E., ZIMM, S., GOEL, R., KIM, S., HORTON, C.M., MCVEY, L., MORRIS, J. & WEISS, R.J. (1990). A phase II trial of intraperitoneal cisplatin and etoposide for primary treatment of ovarian epithelial cancer. *J. Clin. Oncol.*, **8**, 137–154.

KIRMANI, S., ZIMM, S., CLEARY, S.M., HORTON, C. & HOWELL, S.B. (1988). Intraperitoneal cisplatinum and etoposide as salvage therapy for ovarian cancer. *Proc. Am. Soc. Clin. Oncol.*, **7**, 117.

LOS, G. & MCVIE, J.G. (1990). Carboplatin an alternative for intraperitoneal cisplatin treatment in cancers restricted to the peritoneal cavity. *Proc. Am. Soc. Clin. Oncol.*, **7**, 157.

MARKMAN, M., REICHMAN, B., HAKES, T., RUBIN, S., JONES, W., LEWIS, J.L., BARAKAT, R., CURTIN, S., ALMANDRONES, L. & HOSKINS, W. (1992). Phase 2 trial of intraperitoneal carboplatin and etoposide as salvage treatment of advanced epithelial ovarian cancer. *Gynec. Oncol.*, **47**, 353–357.

MEYERS, F.J., WELBORN, J., LEWIS, J.B. & FLYNN, U. (1989). Infusion carboplatin treatment of relapsed and refractory acute leukemia: evidence of efficacy with minimal extramedullary toxicity at intermediate doses. *J. Clin. Oncol.*, **7**, 173–178.

O'DWYER, P.J., LACRETA, F.P., DAUGHERTY, J.P., HOGAN, M., ROSENBLUM, N.G., O'DWYER, J.L. & COMIS, R.L. (1991). Phase I/pharmacokinetic study of intraperitoneal etoposide. *Cancer Res.*, **51**, 2041–2046.

PFEIFLE, C.E., HOWELL, S.B., FELTHOUSE, R.D., WOLIVER, T.B.S., ANDREWS, P.A., MARKMAN, M. & MURPHY, M.P. (1985). High-dose cisplatin with sodium thiosulfate protection. *J. Clin. Oncol.*, **3**, 237–244.

SHEA, T.C., FLAHERTY, M., ELIAS, A., EDER, J.P., ANTMAN, K., BEGY, C., SCHNIPPER, L., FREU III, E. & HENNER, W.D. (1989). A phase I clinical and pharmacokinetic study of carboplatin and autologous bone marrow support. *J. Clin. Oncol.*, **7**, 651–661.

SMITH, I.E., EVANS, B.D., GORE, M.E., VINCENT, M.D., REPETTO, L., YARNOLD, J.R. & FORD, H.T. (1987). Carboplatin (paraplatin; JM8) and etoposide (VP-16) as first-line combination therapy for small-cell lung cancer. *J. Clin. Oncol.*, **5**, 185–189.

SPEYER, J.L., BELLER, U., COLOMBO, N., SORICH, J., WERNZ, J.C., HOCHSTER, H., GREEN, M., PORGES, R., MUGGIA, F.M., CANETTS, R. & BECKMAN, E.M. (1990). Intraperitoneal carboplatin: Favorable results in women with minimal residual ovarian cancer after cisplatin therapy. *J. Clin. Oncol.*, **8**, 1335–1341.

STRIFE, R.J. & JARDIN, I. (1986). Analysis of the anticancer drugs VP16-213 and VM-23 and their metabolites by high-performance liquid chromatography. *J. Chromatogr.*, **182**, 211–220.

TEN BOKKEL HUININK, W.W., VAN DER BURG, M.E.L., VAN OOSTEROM, A.T., NEIJAL, J.P., GEORGE, M., GUASTELLA, J.P., VEENHOF, C.H., ROTMENSZ, N., DALESIO, O. & VERMORKEN, J.B. (1988). Carboplatin in combination therapy for ovarian cancer. *Cancer Treat. Rev.*, **15** (suppl. B) 9–15.

ZIMM, S., CLEARY, S.M., LUCAS, W.E., WEISS, R.J., MARKMAN, M., ANDREWS, P.A., SCHIEFER, M.A., KIM, S., HORTON, C. & HOWELL, S.B. (1987). Phase I/pharmacokinetic study of intraperitoneal cisplatin and etoposide. *Cancer Res.*, **47**, 1712–1716.